# Possible neurotoxicity of the anesthetic propofol: evidence for the inhibition of complex II of the respiratory chain in area CA3 of rat hippocampal slices

**DOI:** 10.1007/s00204-018-2295-8

**Published:** 2018-08-24

**Authors:** Nikolaus Berndt, Jörg Rösner, Rizwan ul Haq, Oliver Kann, Richard Kovács, Hermann-Georg Holzhütter, Claudia Spies, Agustin Liotta

**Affiliations:** 1 Institute of Biochemistry, Charité-Universitätsmedizin Berlin, 10117 Berlin, Germany; 2 Institute for Computational and Imaging Science in Cardiovascular Medicine Charité, Universitätsmedizin Berlin, 13353 Berlin, Germany; 3 Neuroscience Research Center, Corporate Member of Freie Universität Berlin, Humboldt-Universität zu Berlin, and Berlin Institute of Health, Charité-Universitätsmedizin Berlin, 10117 Berlin, Germany; 4 Institute of Physiology and Pathophysiology, University of Heidelberg, 69120 Heidelberg, Germany; 5 Institute for Neurophysiology, Corporate Member of Freie Universität Berlin, Humboldt-Universität zu Berlin, and Berlin Institute of Health, Charité-Universitätsmedizin Berlin, 10117 Berlin, Germany; 6 Department of Anesthesiology and Intensive Care, Corporate member of Freie Universität Berlin, Humboldt-Universität zu Berlin and Berlin Institute of Health, Charité-Universitätsmedizin Berlin, 10117 Berlin, Germany; 7 Berlin Institute of Health, Charité-Universitätsmedizin Berlin, 10117 Berlin, Germany

**Keywords:** Anesthesia, Propofol, Mitochondria, Complex II, Hippocampus, Gamma oscillation

## Abstract

Propofol is the most frequently used intravenous anesthetic for induction and maintenance of anesthesia. Propofol acts first and formost as a GABA_A_-agonist, but effects on other neuronal receptors and voltage-gated ion channels have been described. Besides its direct effect on neurotransmission, propofol-dependent impairment of mitochondrial function in neurons has been suggested to be responsible for neurotoxicity and postoperative brain dysfunction. To clarify the potential neurotoxic effect in more detail, we investigated the effects of propofol on neuronal energy metabolism of hippocampal slices of the stratum pyramidale of area CA3 at different activity states. We combined oxygen-measurements, electrophysiology and flavin adenine dinucleotide (FAD)-imaging with computational modeling to uncover molecular targets in mitochondrial energy metabolism that are directly inhibited by propofol. We found that high concentrations of propofol (100 µM) significantly decrease population spikes, paired pulse ratio, the cerebral metabolic rate of oxygen consumption (CMRO_2_), frequency and power of gamma oscillations and increase FAD-oxidation. Model-based simulation of mitochondrial FAD redox state at inhibition of different respiratory chain (RC) complexes and the pyruvate-dehydrogenase show that the alterations in FAD-autofluorescence during propofol administration can be explained with a strong direct inhibition of the complex II (cxII) of the RC. While this inhibition may not affect ATP availability under normal conditions, it may have an impact at high energy demand. Our data support the notion that propofol may lead to neurotoxicity and neuronal dysfunction by directly affecting the energy metabolism in neurons.

**Electronic supplementary material:**

The online version of this article (10.1007/s00204-018-2295-8) contains supplementary material, which is available to authorized users.

## Introduction

Propofol is the most frequently used intravenous anesthetic for sedation or total intravenous anesthesia during surgery and intensive care treatment (McKeage and Perry 2003). The β-subunit of the GABA_A_-receptor is considered to be the principal molecular target of propofol (Yip et al. 2013; Shin et al. 2018), but reduction of neuronal transmission through blockade of Na^+^-channels, nicotinic-receptors, glutamate-receptors and decrease in presynaptic transmitter release has been described as well (Martella et al. 2005; Kajimoto et al. 2014; Bademosi et al. 2018). Besides its direct effect on neurotransmission, propofol affects mitochondrial function in neurons (Bains et al. 2009; Marian et al. 1997; Wang et al. 2016 among others) which may trigger neurotoxicity and neuronal loss in the immature central nervous system (CNS) (Twaroski et al. 2015; Kajimoto et al. 2014).

The brain is one of the most energetically expensive organs in the vertebrate body receiving about 20% of cardiac output (Kety 1950). Brain energy metabolism relies predominantly on the degradation of glucose (and lactate) to pyruvate and its subsequent oxidation in the mitochondrial citric acid cycle (CAC). Thus, the majority of the cellular adenosine triphosphate (ATP) needed for proper brain functionality is generated in the mitochondria via oxidative phosphorylation (Rolfe and Brown 1997).

Bains et al. demonstrated that propofol can lower ATP-production by mitochondrial depolarization due to inhibition of Complex I–IV of the RC in isolated synaptosomes (Bains et al. 2009). In immature swine, propofol anesthesia provokes a hypoxic phenotype characterized by glycogen and ATP-depletion due to disturbances in the CAC intermediates and changes in flux distribution in the RC (Kajimoto et al. 2014). Furthermore, the systemic metabolic disorder called propofol infusion syndrome may unmask preexisting functional deficiency in the RC complexes (Finsterer and Frank 2016). In view of the multiplicity of propofol effects in the CNS, it remains controversial whether the influence of propofol on the oxidative phosphorylation may protect or harm neurons. Since millions of patients receive propofol each year, the influence on mitochondrial function as a possible pathomechanism for postoperative brain dysfunction (i.e. postoperative delirium or postoperative cognitive dysfunction) needs further clarification.

The aim of this study was to assess direct effects of propofol on mitochondria and to characterize the possible functional consequences for neurons. To this end we used brain slices from area CA3 of the hippocampal formation and studied propofol-induced alterations in the energy metabolism at basal activity and increased activity during gamma oscillations. Gamma oscillations are an established in vitro model for network activity associated with high cognitive functions and increased energy demand (Bartos et al. 2007; Huchzermeyer et al. 2008). In our approach, we combined measurements of tissue oxygen (pO_2_), local field potentials (f.p.), extracellular potassium ([K^+^]_o_) and FAD-autofluorescence with computer modeling. All measurements were performed in the area CA3 of the hippocampal formation since this area plays an important role in the generation of network oscillations relevant for cognition, spatial exploration and memory formation (Eichenbaum 2017).

In order to identify the precise mode of action of propofol on mitochondria we took advantage of the fact that the fluorescence emission of the flavine nucleotide FAD depends on its redox state. The oxidized form of FAD contains an isoalloxazine chromophore which is fluorescent when excited with blue light, whereas FADH_2_ is not fluorescent. Temporal changes in the redox state of FAD are mainly caused by redox reactions of mitochondria, as FAD is a prosthetic group of the pyruvate dehydrogenase complex (PDHC), the *α*-ketogluterate dehydrogenase complex (KGDHC), the glycerol-3-phosphate dehydrogenase (G3PDH) and the succinate dehydrogenase (SUCCDH) (Berndt et al. 2015). Hence, monitoring FAD-autofluorescence allows a direct measurement of changes in mitochondrial redox state (Scholz et al. 1969; Rösner et al. 2016). Computer simulations of cellular metabolism allow to assess changes in metabolites and the rates of their mutual chemical interconversion in response to varying external conditions (Berndt and Holzhütter 2016). In our approach, the combination of experimental data with computer modeling of metabolic processes allows to discriminate between different putative targets of propofol in mitochondrial oxidative phosphorylation and to quantify the contribution of lowered neuronal activity and direct inhibition of mitochondrial ATP production on the lowered energy demand of neuronal tissue in the presence of propofol.

## Materials and methods

### Slice preparation and maintenance

For this study, 44 adult male Wistar rats (weight 200–250 g, age 6–8 weeks) were killed in accordance with the Helsinki declaration and institutional guidelines (LAGeSo, T0096/02). Animals were decapitated under anesthesia with isoflurane (2%) and laughing gas (N_2_O, 70%). Brains were rapidly removed and transferred to cold and gassed (carbogen, 95% O_2_ and 5% CO_2_) artificial cerebrospinal fluid (aCSF) containing (in mM): 129 NaCl, 21 NaHCO_3_, 10 glucose, 3 KCl, 1.25 NaH_2_PO_4_, 1.6 CaCl_2_ and 1.8 MgCl_2_. Osmolarity and pH were 295–305 mosm/L and 7.35–7.45, respectively. Horizontal hippocampal slices (400 µm thick) were prepared with a Leica VT 1200 S vibratome (Leica, Wetzlar, Germany) and stored in an interface chamber with continuous aCSF perfusion (flow rate of 2 mL/min, temperature 34–35 °C, gassed with carbogen). Experiments started after 2 h of recovery following the slicing procedure. For FAD-fluorescence imaging, the slices were transferred to a submerged chamber (flow rate 10 mL/min, temperature ca. 34–35 °C) following the recovery period.

### Electrophysiology, oxygen recordings and fluorescence recordings

**Fig. 1:**
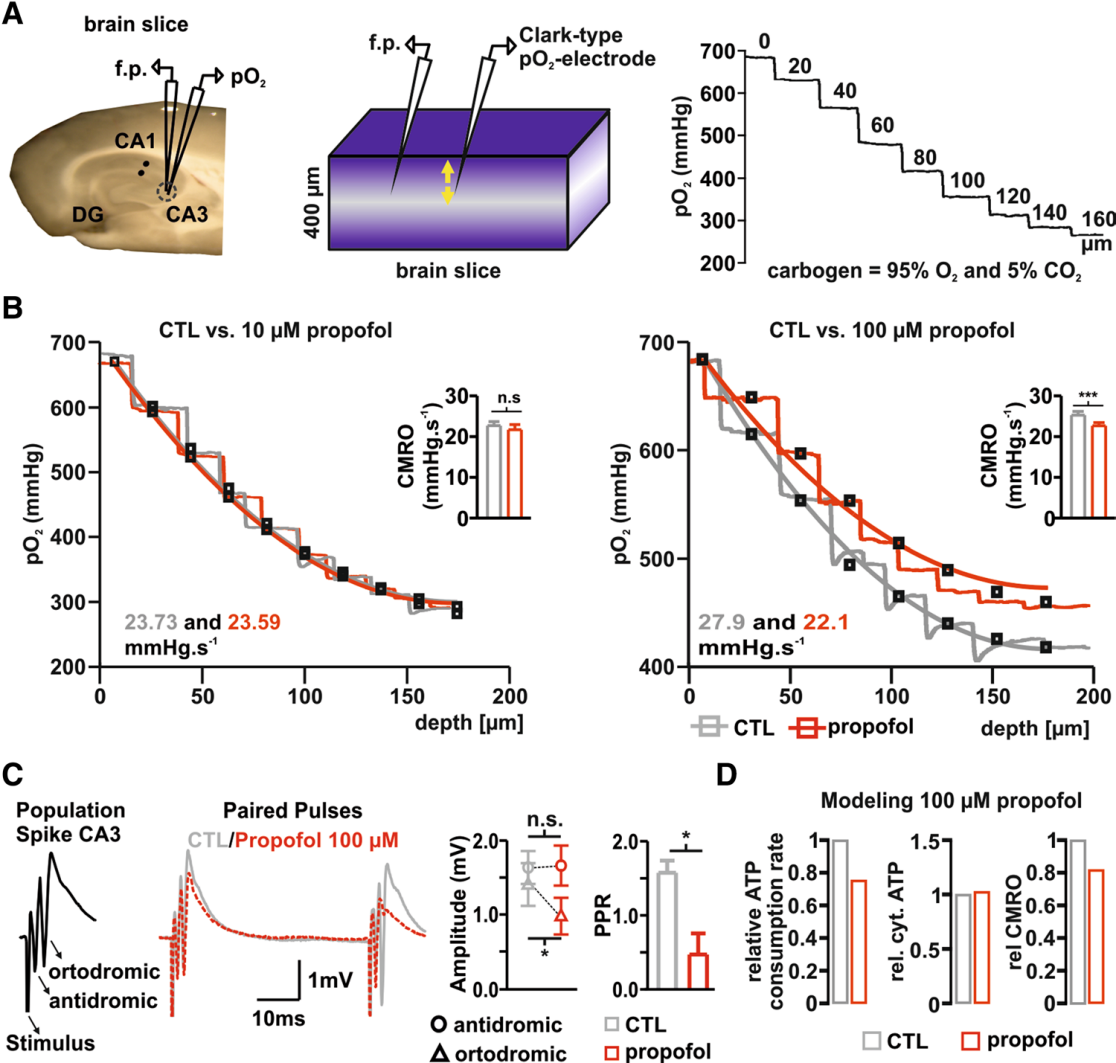
Propofol reduction of CMRO_2_ is associated with impaired synaptic transmission in hippocampal slices. **a** Left, picture of hippocampal slice and sketch of typical electrodes positioning during simultaneous field potential and pO_2_ recordings in the stratum pyramidale of area CA3. Middle: representation of brain slice under interface conditions. The tissue is provided with carbogen from surface and bottom and pO_2_ decreases with the distance to the source of oxygen (i.e. distance to the slice bottom and surface, fading blue) providing typical depth profiles (see right, numbers in trace—distance to slice surface in µm). The oxygen gradient depends on oxygen supply, solubility (constant under experimental conditions) and cellular respiration. Simultaneously to the pO_2_-measurements, changes in synaptic transmission were assessed. For this purpose, the Schaffer collaterals were electrically stimulated in the stratum radiatum of area CA1 (in the slice-picture, black dots) while population spikes were obtained with a glass microelectrode from CA3 in the vicinity of the oxygen probe. **b** Examples traces of 20 µm O_2_-steps measured before (control (CTL), gray traces) and during treatment with 10 µM and 100 µM Propofol (red traces, left and right respectively). Using a reaction–diffusion model, CMRO_2_ was calculated (gray and red curves for control and propofol, respectively). While the application of 10 µM Propofol did not altered respiration, 100 µM Propofol significantly decreased CMRO_2_ (left and right quantitative analysis plots, respectively). **c** Changes in orthodromic, antidromic PS and PPR after treatment with 100 µM propofol (traces: gray control-condition and red Propofol-condition). Left: example PS of area CA3 consisting of an antidromic and orthodromic PS after electrical stimulation. Middle: overlay of PP during control (CTL: gray trace) and after treatment with 100 µM propofol (red dotted trace). As plotted on the right: the orthodromic PS and PPR significantly decreased indicating reduction in synaptic transmission and probability of presynaptic transmitter release. **d** Plots of computed relative changes in ATP and CMRO_2_ during treatment with 100 µM Propofol. Left: ATP consumption decreases by ~ 25%. Middle: cytosolic ATP levels increased from 3.08 mM to 3.16 mM, while oxygen consumption rate decreased to ~ 82% (left). All plots display Mean + SEM, significance was tested using paired *t* tests, *n.s*. non significant, * < 0.05, **0.01 and ***0.001

All experiments were performed in the stratum pyramidale of area CA3 of the hippocampal formation while electrical stimulation (if applicable) was applied with a bipolar electrode (platinum, 20 µm) in the stratum radiatum of area CA1 (see also Fig. 1a). Electrical stimuli applied to the Schaffer collateral (SC) in CA1 induces an antidromic population spike (PS) followed by an orthodromic PS in area CA3. The amplitude of the antidromic PS is relative to the number of activated fibers in the presynapse and is tetrodotoxin (TTX)-sensitive. The antidromic activation of pyramidal cells leads to the generation of a secondary orthodromic PS, which reflects the postsynaptic activation of pyramidal cells after activation of recurrent axons within the CA3 network itself (Çalışkan et al. 2015). Glass microelectrodes filled with saline (154 mM NaCl) were used for f.p. recordings. Stimulation consisted in 2 single pulses of 100 µs duration with an interval of 50 ms repeated 5 times for each experimental condition (paired pulse). To induce FAD, O_2_ and [K^+^]_o_ transients, 2 s long 20 Hz tetani were applied (single pulse 100 µs duration, interval 50 ms, 40 pulses) every 10 min as previously described (Rösner et al. 2016). Pulse generation was performed with Master 8 (A.M.P.I., Jerusalem, Israel). Simultaneous f.p. and [K^+^]_o_ measurements were performed using double-barreled ion-sensitive microelectrodes in experiments concerning simultaneous recordings of FAD, pO_2_ and [K^+^]_o_ during stimulus-induced transients. The reference side of the electrode was filled with 154 mM NaCl while the ion-sensitive side was filled with 100 mM KCl and its tip contained potassium ionophore I 60,031 (Fluka, Sigma, Buchs, Switzerland). We than calculated [K^+^]_o_ using a modified Nernst’s equation as described previously (Liotta et al. 2012). Partial oxygen pressure (pO_2_) was measured using Clark-style oxygen sensors (tip: 10 µm; Unisense, Aarhus, Denmark) placed near the field electrode or the ion-sensitive microelectrode. Oxygen electrodes were polarized for > 12 h and two-point calibrated in aCSF gassed with 50% and 95% O_2_ at 35 °C before each recording session. For depth profiles, the pO_2_-electrode was fixed to a mechanical manipulator (Narishige, Japan) and moved vertically through the slice in steps of 20 µm until additional steps no longer reduced pO_2_ (usually ~ 200 µm below surface, see also Fig. 1). FAD-autofluorescence was recorded under submerged conditions using a custom-built imaging setup equipped with a light emitting diode (LED, 460 nm wavelength) and a photomultiplier tube (PMT). For this purpose, a X20 submerged objective was focused on stratum pyramidale of area CA3 to obtain autofluorescence from neuron’s somata (Zeiss, Oberkochen, Germany).The LED (Lumen, Prior scientific, Seefelder, Germany) was set at 18% intensity (Power in focus plane with objective: 2.390 mW) and was triggered externally with a Master 8 (A.M.P.I., Jerusalem, Israel). To reduce bleaching and phototoxicity we performed excitation with pulsed light (5 ms, 5 Hz) as described in (Rösner et al. 2016).

### Drugs

**Fig. 2:**
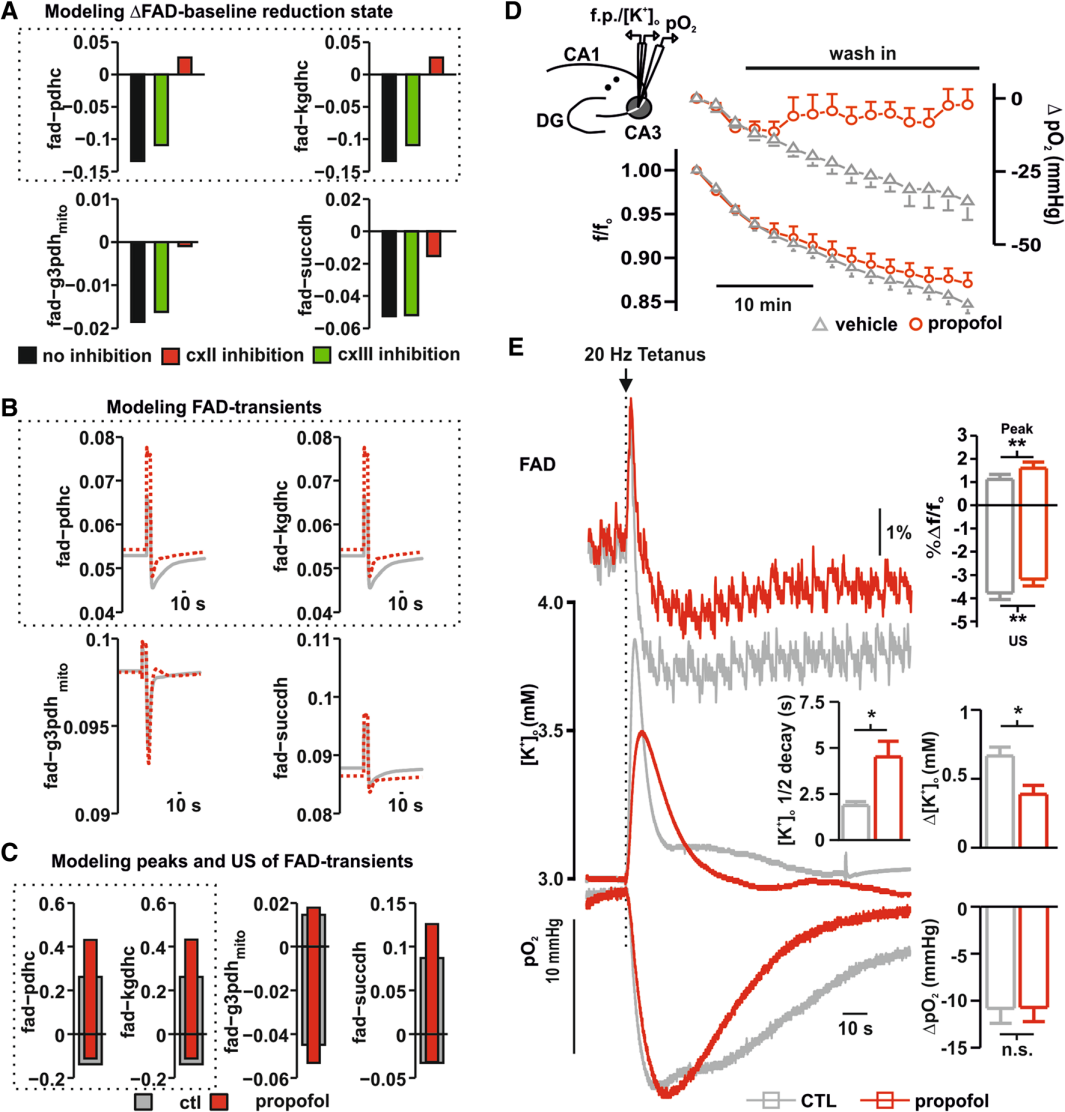
Computational modeling and effects of propofol on FAD, extracellular [K^+^]_o_-rises and pO_2_-consumption in area CA3. **a** Simulated effects of propofol on FAD-baseline changes in the PDHC, KGDHC, G3PDH_mito_ and SUCCDH for three possible effects. Black: no direct metabolic effect of propofol; Green: direct inhibition of cxIII by propofol; Red: direct inhibition of cxII by propofol. **b** Simulated stimulus-induced FAD-transients of PDHC, KGDHC, G3PDH and SUCCDH. Gray line shows FAD transients for CTL conditions; red lines depict FAD transients for propofol conditions when a cxII-inhibition was assumed. **c** Simulated peak and undershoot of FAD-transients of PDHC, KGDHC, G3PDH and SUCCDH. Gray bars correspond to CTL conditions; red bars apply for propofol conditions assuming cxII-Inhibtion. **d** Overlay of oxygen and FAD-baseline during application of propofol (red) or vehicle containing DMSO 0.1% (gray). Importantly, 100 µM propofol provoked a shift to oxidation in FAD. **e** Exemplary recording of simultaneous measurements of stimulus-induced FAD-transients (Top), [K^+^]_o_-rises (middle) and O_2_-decays (bottom) performed in the area CA3, while stimulating the stratum radiatum in area CA1 as shown in the graphical representation (see **d** left on the top). FAD-transients display a stimulus (20 Hz, 2 s, arrow) induced biphasic response: a sharp peak (oxidation phase) followed by a long-lasting undershoot (reduction phase). Compared to the control situation (gray trace), after application of 100 µM Propofol (red trace) the redox state was significantly shifted to oxidation as shown in the statistics (plot on left). Simultaneously recorded [K^+^]_o_-rises (middle, control gray traces and 100 µM Propofol red traces) display a significant decrease in tissue excitability in the presence of high concentrations of propofol (see also statistics plot, control gray, propofol red). Importantly, the half-decay time was significantly larger under propofol. The corresponding O_2_-decays (bottom) did not undergo significant changes when comparing the control situation (gray traces) with the treatment with propofol (red traces) although baseline pO_2_ was increased under propofol. All plots display Mean + SEM, significance was tested using paired *t* tests, *n.s*. non significant, *< 0.05, **0.01 and ***0.001

Bath application of drugs dissolved in aCSF was performed to test propofol effects. Propofol (2,6-diisopropylphenol) was dissolved in DMSO (maximal final concentration of DMSO was 0.1% in the solutions) and applied during ca. 20 or 45 min in the submerged or interface system, respectively. Since propofol is an allosteric agonist of GABA_A_ receptors (Shin et al. 2018), we performed experiments concerning effects of propofol on CMRO_2_ and FAD in the absence of GABAergic transmission to test independence of metabolic and synaptic effects. For this purpose, slices were treated with the GABA_A_ antagonist bicuculline (10 µM) prior to propofol treatment. Since the application of bicuculline results in the generation of strong glutamatergic input and epileptiform discharges in area CA3 (data not shown, see our prior works Liotta et al. 2011; Schoknecht et al. 2017), a set of supplementary experiments were performed using a cocktail containing bicucculine (10 µM), DL-amino-5-phosphonopentanoic acid (AP-5, 50 µM) and 6-cyano-7-nitroquinoxaline-2,3-dione (CNQX, 50 µM) to block both GABAergic and glutamatergic input onto CA3 pyramidal cells (see Fig. 4). Pharmacological induction of gamma oscillations was achieved with 10 µM acetylcholine chloride and 2 µM Physostigmine salicylate applied during ca. 90 min until stable gamma oscillations (stable frequency and power in online sonogram during more than 15 min) developed. We performed a group of experiments to exclude metabolic effects of DMSO; for this purpose, aCSF containing 0.1% DMSO was applied after 10 min baseline recording for 20 min. As shown in Fig. 2d and supp. Figure 1, FAD-Baseline, FAD-Transients and electrophysiology signals (PS and PPR) remained almost unchanged in the presence of DMSO (*n* = 5, *N* = 4).

### Data acquisition and data analysis

Analog signals were digitalized with Power1401 and recorded with Spike2 (Cambridge Electronic Design Limited, Cambridge, UK). Data analysis and statistics were performed using Spike2, Origin software (Version 6, Microcal Software, Northampton, MA, USA) and SPSS v.20.0 (IBM Corporation, Armonk, NY, USA). Absolute PS amplitude was measured using the cursor measurement function of Spike2. If not described otherwise, we plotted and described the Mean ± standard error of mean (SEM). Paired pulse ratio (PPR) was calculated by dividing the amplitude of the 2nd orthodromic PS through the 1st orhtodromic PS. Fluorescence is shown as *Δf*/*f*_o_ where *f*_*0*_ is the baseline fluorescence intensity (average of 15 s baseline prior to stimulation in the case of transients or baseline recording). The absolute [K^+^]_o_ was calculated using a modified Nernst equation and the half-time decay was measured using the cursor functions of Spike2. Power spectra of gamma oscillations were analyzed with a Spike2 by fast Fourier transformation (Hanning window, FFT size 4096). For statistical inference, we performed paired Student’s *t* tests (with Bonferroni correction when necessary) or Wilcoxon signed rank test depending on distribution. For the number of experiments, “*n*” refers to the number of slices and “*N*” to the number of animals used for the experimental set. Experiments were performed in slices prepared at least from three different animals. Changes were stipulated to be significant for *p* < 0.05.

### Calculation of basal oxygen consumption rates

Cerebral metabolic rate of oxygen (CMRO_2_) was calculated from pO_2_ depth profiles measured under interface conditions as described in (Huchzermeyer et al. 2013). In short, we applied a reaction–diffusion model for oxygen consisting of diffusive oxygen transport and oxygen consumption within the slice. The slices were divided into layers with equal thickness of 1 µm. Diffusive distribution of oxygen between the layers is described by Fick’s Law with a diffusion constant of 1.6 × 10^3^ µm^2^/s and oxygen consumption rate within each layer is given by Michaelis–Menten kinetics with a *K*_m_-value of 3 mmHg (Kasischke et al. 2011). The CMRO_2_ was assumed to be homogeneous throughout the slice (i.e., equal in every layer) and is treated as adjustable parameter to match the experimental data. Dirichlet boundary conditions were used at the slice surface and Neumann boundary conditions were used at the pO_2_ minimum.

### Calculating of FAD transients and ATP consumption rates

We used a metabolic model of neuronal energy metabolism (Berndt et al. 2015) to simulate stimulus-induced FAD transients and ATP consumption rates. Using the CMRO_2_s obtained from pO_2_ measurements under interface conditions and assuming feasible cytosolic calcium transients, we determined ATP consumption rates for the different conditions using the maximal ATP consumption rate as adjustable parameter. For all simulations we used MATLAB Release2012a (The MathWorks, Inc., Natick, MA, USA) with the optimization tool box.

## Results

### Effects of propofol on CMRO_2_ and synaptic transmission

First, we quantified the effect of propofol on neuronal energy metabolism in our standard experimental conditions, i.e. during spontaneous asynchronous neuronal network activity (Martella et al. 2005). pO_2_ depth profiles were recorded in the stratum pyramidale of area CA3 in hippocampal slices with an approximate thickness of 400 µm under interface conditions at 95% oxygenation (Fig. 1a, see also “Materials and methods”). This gave maximal pO_2_ values of ~ 680 mmHg at the slice surface, which declined to ~ 350 mmHg at the slice core before rising again in the lower third of the slice as oxygen diffused from the bottom surface of the slice via the aCSF. Depth profiles were recorded under control conditions (aCSF only) and in the presence of 10 and 100 µM propofol (Fig. 1a, b). We used a mathematical reaction–diffusion model (Huchzermeyer et al. 2013) to calculate the CMRO_2_ from the experimentally determined pO_2_ depth profiles (Fig. 1). The CMRO_2_ was unchanged with 10 µM propofol (control 24.04 ± 0.4 vs. 23.22 ± 0.9 mmHg/s after application of 10 µM propofol, *n* = 6, *N* = 3, *p* = 0.44, paired t-test), but decreased significantly from 25.2 ± 0.9 mmHg/s under control conditions to 22.6 ± 0.8 mmHg/s in the presence of 100 µM propofol (*n* = 13, *N* = 6, *p* = 0.02, Wilcoxon). Next, we used the CMRO_2_ determined for control and propofol conditions to simulate ATP consumption rates similar as in (Schoknecht et al. 2017). These simulations revealed a decrease of 30% in basal ATP demand accompanied by an increase of 0.1 mM in ATP content during 100 µM propofol administration (Fig. 1d).

High concentrations of propofol also affected synaptic transmission and probability of transmitter release—as can been seen in Fig. 1c showing stimulus-induced PS and PPR with and without propofol. Stimulus-induced PS in the Stratum pyramidale of area CA3 has characteristically a first antidromic PS and a second orthodromic PS when the Schaffer collaterals are electrically stimulated in the Stratum radiatum of area CA1. The antidromic PS is tetrodotoxin sensible and underlies retrograde and passive axonal transmission to the soma of CA3 pyramidal cells (Liotta et al. 2012). The orthodromic PS takes place after the first PS via activation of Schaffer collaterals and recurrent synaptic activation of the pyramidal cells (Çalışkan et al. 2015). When applied in low concentrations (10 µM propofol), neither the antidromic nor the orthodromic response changed significantly (antidromic PS 1.99 ± 0.2 in the control vs. 2.11 ± 0.1 mV under propofol, *p* = 0.27 and orthodromic PS 2.48 ± 0.8 in the control vs. 2.27 ± 0.34 mV under propofol, *p* = 0.72, *n* = 8, *N* = 3, paired *t* test). Similarly, the PPR did not display significant changes as well (1.6 vs. 1.58 in control and under propofol, respectively, *p* = 0.83, *n* = 8, *N* = 3, paired *t* test). As expected and in line with the changes in CMRO_2_, the application of 100 µM propofol was associated with stable antidromic PS (1.63 ± 0.22 vs. 1.66 ± 0.26 mV for control and under propofol respectively, *p* = 0.8, *n* = 8, *N* = 4, paired *t* test) while the orthodromic PS and PPR decreased significantly (orthodromic PS 1.4 ± 0.28 vs. 0.98 ± 0.25 mV, *p* = 0.04, and PPR: 1.57 ± 0.16 vs. 0.47 ± 0.29, p = 0.002, for control and propofol respectively, *n* = 8, *N* = 4, paired *t* test). Since propofol reduced orthodromic PS and PPR, a reduction of synaptic transmission would explain the reduced energy demand. The question arises if the decrease in CMRO_2_ observed during propofol administration is a mere consequence of lowered ATP consumption rate due to (partial) blockade of synaptic processes or if a direct effect of propofol on neuronal energy metabolism must be considered. To test this, in a supplementary set of experiments we measured CMRO_2_ in the presence of the GABA_A_-antagonist bicuculline (10 µM) or a cocktail containing AP-5 (50 µM), CNQX (25 µM) and bicculline (10 µM) to block both GABAergic and glutamatergic postsynaptic transmission. Under both conditions, the application of 100 µM propofol significantly reduced CMRO_2_, demonstrating a similar decrease in metabolism in the absence of GABAergic and glutamatergic transmission. In slices pre-treated with 10 µM bicuculline, CMRO_2_ significantly decreased from 26.68 ± 3.7 to 20.76 ± 1.7 mmHg/s when 100 µM propofol was added (CMRO_2_ prior to bicuculline was 22.25 ± 1.8 mmHg/s, *n* = 8, *N* = 3, paired *t* test). Slices treated with bicuculline displayed stimulus-induced and spontaneous recurrent epileptiform discharges and treatment with 100 µM propofol slightly impaired amplitude and incidence of these events (data not shown). To check if propofol just decreased CMRO_2_ by inhibition of spontaneous recurrent discharges, we applied the cocktail to exclude this possibility. Interestingly, in slices treated with the cocktail, the CMRO_2_ under control conditions did not significantly change, but decreased in the presence of propofol (control: 27.03 ± 2.7 and with the cocktail CMRO_2_ was 26.0 ± 2.7 vs. 22.12 ± 2.2 after 100 µM propofol, *p* = 0.6 and *p* = 0.004 respectively, *n* = 7, *N* = 3, paired *t* test). The fact that basal oxygen consumption did not change after the application of the cocktail was surprising and reinforced the idea of a strong, synaptic-independent, effect of propofol in mitochondrial function.

### Computational modeling, effects on FAD and simultaneous changes in synaptic transmission

In the literature, the list of putative effects of propofol on synaptic transmission and mitochondrial function is long and the resulting consequences for neuron’s homeostasis are unknown. Mitochondrial depolarization and inhibition of complex I to IV of the RC have been described in vitro (Bains et al. 2009; Bergamini et al. 2016 among others). In most of the cases, the experiments were performed in isolated preparations, whereas in (Kajimoto et al. 2014) it was shown that propofol impedes the electron flow through the RC and coenzyme Q is the main site of interaction with propofol in the brain of immature swine under anesthesia. The authors of this relevant in vivo study also concluded that complex I is not the molecular target of propofol, thus leaving complex II (cxII) and complex III (cxIII) as possible relevant sites of propofol action on the RC in vivo. Depending on the molecular target of propofol within the RC, mitochondrial redox state would show characteristic changes in the different scenarios. As the mitochondrial redox state is adequately reflected by FAD autofluorescence (Shuttleworth 2010), we used this signal to discriminate the possible molecular targets of propofol in the RC. Therefore, we simulated basal FAD-changes and stimulus-induced FAD-transients taking into account the measured decrease in CMRO_2_ (see Fig. 1) under the assumption that propofol (1) has no direct effect on mitochondrial energy metabolism, (2) directly inhibits cxII or (3) directly inhibits cxIII of the RC (Fig. 2). For these three scenarios, the simulated FAD profiles were compared with FAD autofluorescence recordings in brain slices. The simulations in Fig. 2a show that assuming either no direct inhibitory effect of propofol or a cxIII-inhibition (75%) results in a marked decrease of the baseline FAD-autofluorescence (reduction shift) when we used in the simulation the calculated reduction in the ATP consumption rate at 100 µM propofol (see Fig. 1). Contrarily, assuming a direct inhibitory effect (75%) on the on the SUCCDH (cxII) resulted in minimal reduction of FAD associated with G3PDH and SUCCDH and marked oxidation of FAD coupled to PDHC and KGDHC. Comparison of the simulated FAD redox states arising from reduced neuronal ATP consumption rate with experimentally determined FAD-autofluorescence during propofol administration (Fig. 2d) shows that the experimentally observed increase in FAD oxidation is in concordance with the simulation of a cxII-inhibition (see also Fig. 4). In these recordings, application of 100 µM propofol caused a non significant but slight oxidation shift of the baseline compared with control measurements with the vehicle (20 min after wash in the overall f/f_o_ decay were 0.84 vs. 0.87 for control measurements and propofol respectively) while, as in the recordings concerning CMRO_2_-changes, punctual pO_2_-measurements displayed decrease in cellular respiration (Fig. 2d).

**Fig. 3:**
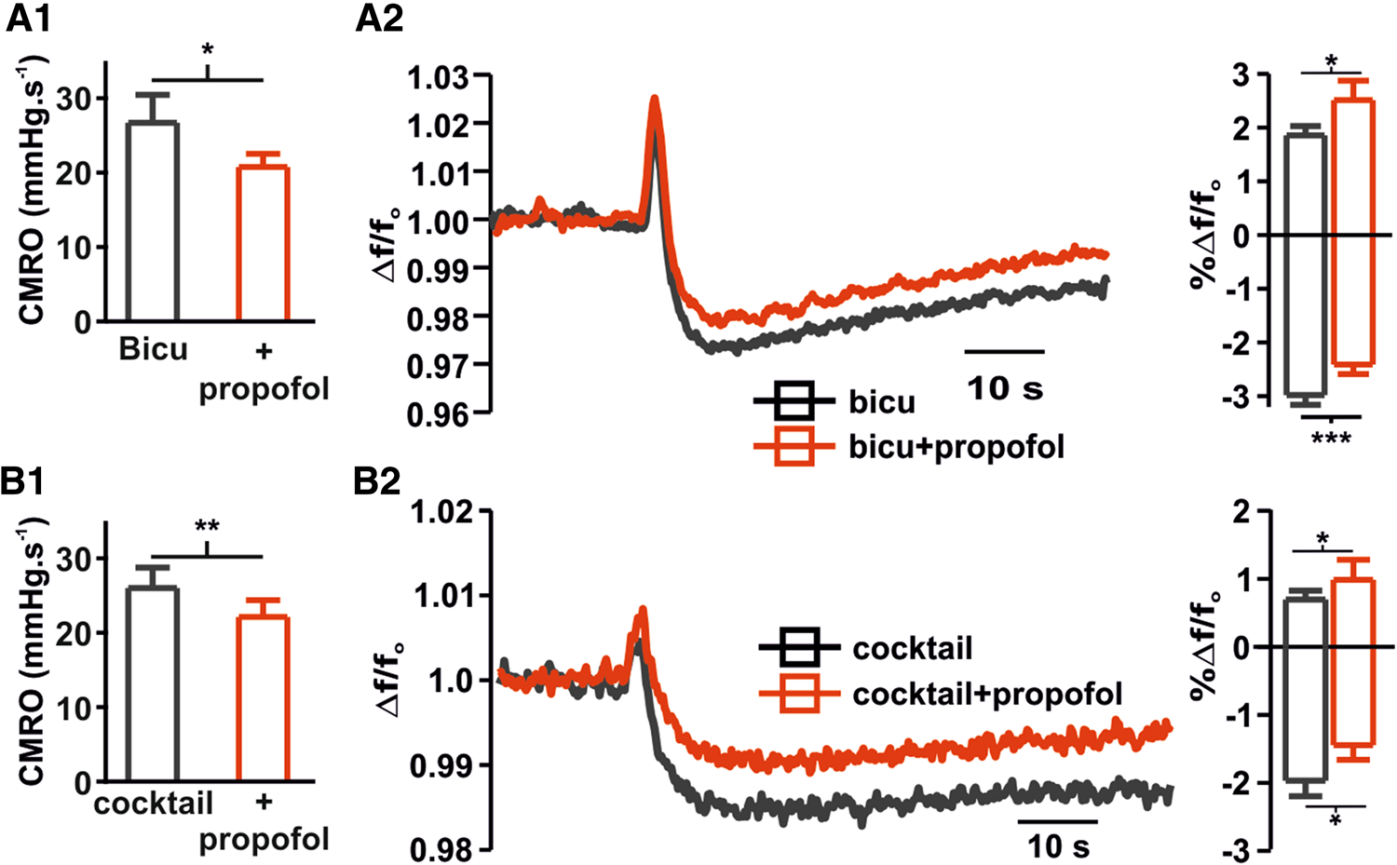
Propofol-induced changes in CMRO_2_ and FAD redox state in the absense of GABAergic and glutamatergic transmission.** A1**,** A2** Since propofol is known to act in GABA_A_-receptors, we checked the effects in CMRO_2_ and FAD after blockade of GABAergic input onto CA3 pyramidal cells with 10 µM Bicuculline (Bicu, gray). The application of 100 µM propofol decrease CMRO_2_ and generated an increment of in FAD oxidation (right: average FAD-signal of recorded transient and left plots of changes in peak and undershoot of transients) independently of GABAergic transmission.** B1**,** B2** The absence of GABAergic transmission is related to increase in glutamatergic excitation and epileptiform discharges (not shown). By pharmacological blockade of both, the GABAergic and glutamatergic input onto CA3-neurons we aim to further differentiate a direct effect of propofol on CMRO_2_ and FAD. Interestingly, 100 µM propofol decreases oxygen consumption and increases FAD-oxidation as well demonstrating a robust effect in neuronal metabolism (right: average FAD-signal of recorded transient and left plots of changes in peak and undershoot of transients). All plots display mean + SEM, significance was tested using paired *t* tests, *n.s*. non significant, *< 0.05, **0.01 and ***0.001

To further substantiate the proposed effect of propofol on cxII, we simulated the effect of a sudden increase in ATP demand on neuronal FAD-autofluorescence with and without propofol (Fig. 2b, c). The increase of the ATP demand corresponds to a sudden activating stimulus (Berndt et al. 2015) and can be used in the experimental setting to study FAD during different metabolic situations (i.e. stimulus induced FAD-transients, see also Rösner et al. 2016). Analogous to the prior simulations, modeled stimulus-induced FAD-transients predicted an upward shift of the peak and lowered magnitude of the undershoot in response to an increase in energy demand during cxII-inhibition (Fig. 2b, c). Comparing the simulated stimulus-induced FAD fluorescence changes during propofol administration with the experimentally determined values (Fig. 2b–e) confirms the predicted oxidation shift (*f*/*f*_o_ oxidation peak increase from 1.11 ± 0.2 to 1.59 ± 0.24 and reduction undershoot decreases from − 3.76 ± 0.26 to − 3.17 ± 0.27 for control and 100 µM propofol, respectively, *p* = 0.005 and 0.01, respectively, *n* = 8, *N* = 4, paired *t* test). In these recordings, the simultaneously measured stimulus-induced [K^+^]_o_-rise was decreased as a sign of impaired synaptic transmission and decreased excitability (Δ[K^+^]_o_ decreased from 0.66 ± 0.06 to 0.38 ± 0.06 mM for control and after 100 µM propofol administration, respectively, *p* < 0.001, *n* = 8, *N* = 4, Fig. 2e, middle trace, paired *t* test). Interestingly, 100 µM propofol also impaired potassium homeostasis as recovery to baseline after tetanus was delayed implying a compromise of ATP-dependent processes (half-decay time in control 1.8 ± 0.2 s vs. 4.5 ± 0.8 s under 100 µm propofol, *p* = 0.004, *n* = 8, *N* = 4, paired *t* test). Simultaneously recorded stimulus-induced drop in partial oxygen pressure showed that the metabolic response was indeed unaltered (Δ pO_2_ 10.8 and 10.7 mmHg for the control and after 100 µM propofol, respectively, *p* = 0.91, paired *t* test, *n* = 8, Fig. 2e) while, as described before, baseline cellular respiration decreased (Fig. 2d). To omit the possibility that the observed changes in FAD-autofluorescence are only a consequence of decreased synaptic transmission, FAD-transients were measured after application of propofol under blockade of the GABAergic and both GABAergic and glutamatergic transmission. As in the experiments in naïve slices and in line with the observed changes in CMRO_2_, 100 µM propofol shifted FAD-transients to oxidation in the presence of bicuculline or the cocktail containing AP-5, CNQX and bicuculline (Fig. 3). Thus, propofol-induced changes in FAD signal are present independently of the main excitatory and inhibitory postsynaptic input onto CA3 pyramidal cells.

**Fig. 4:**
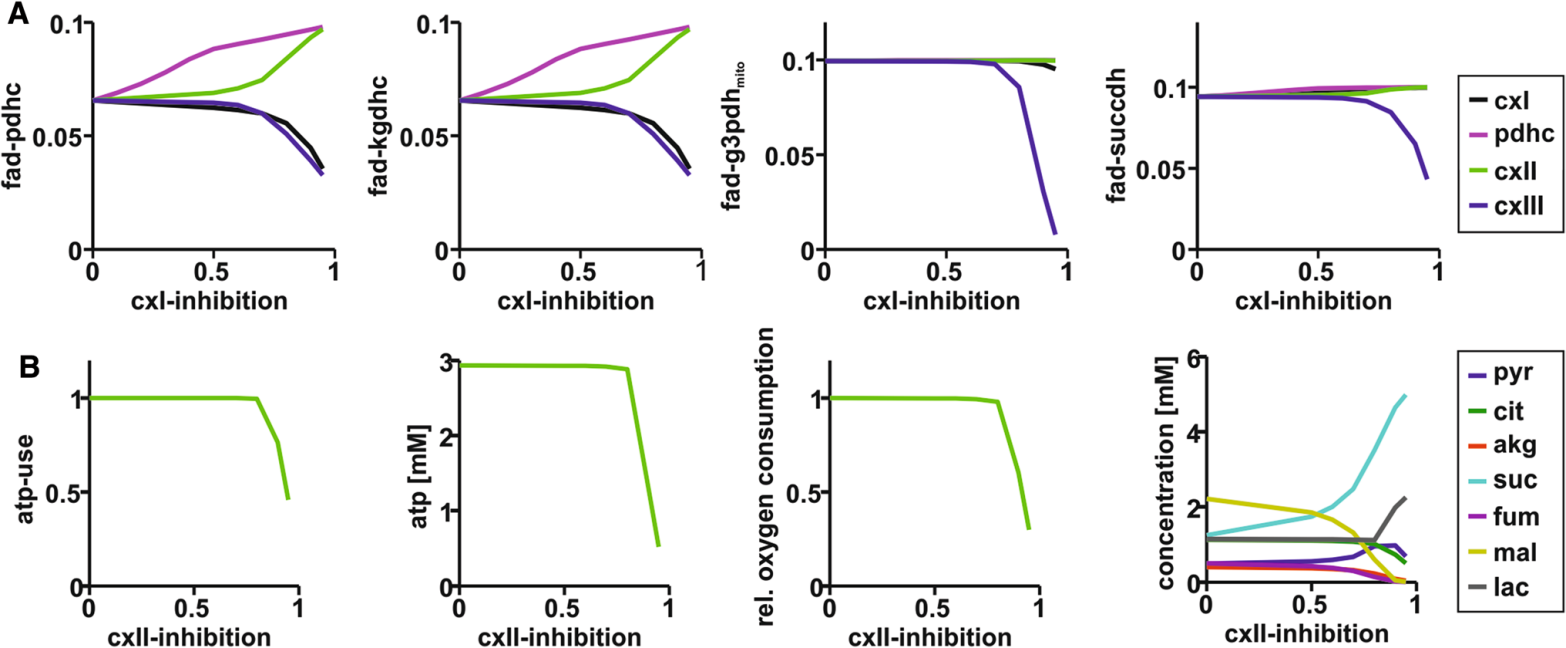
Computational modeling of enzyme inhibition on FAD redox state and neuronal energy metabolism. **a** Simulated changes in FAD-autofluorescence for PDHC, cxI, cxII and cxIII inhibition. Importantly, oxidation of FAD-autofluorescence as found in our experiments (Fig. 2d) can be observed only in PDHC and cxII-inhibition. **b** Simulation of the effect of cxII-inhibition on ATP consumption rate, neuronal ATP content, relative CMRO_2_, neuronal metabolite concentrations. The model predicts a stable mitochondrial function without critical changes until ~ 80% of the SUCCDH is blocked

To examine the FAD-autofluorescence changes in dependence of the degree of enzyme inhibition, we used the metabolic model to investigate the effect of increasing inhibition of PDHC, cxI, cxII and cxIII on neuronal energy metabolism by stepwise decreasing activity levels (*V*max-values) from 100% (no inhibition) to 5% (0.95 inhibition). Figure 4a shows the simulated FAD reduction states in dependence of enzyme inhibition. Only PDHC and cxII inhibition are in concordance with the tendencies in the observed FAD autofluorescence. However, while PDHC inhibition leads to an FAD oxidation shift already at minor inhibition, cxII inhibition displays a lag-phase, where FAD redox state remains almost constant. Thus, only cxII inhibition accounts for the observed oxidative shift in FAD signaling at high (100 µM) propofol concentration. Figure 4b shows the relative ATP consumption rate, cytosolic ATP levels, relative oxygen consumption rate, change of metabolite concentrations and FAD reduction states in dependence of cxII-inhibition. While cx-II-inhibition up to ~ 75% does not affect ATP availability, respiration rate and ATP consumption, there is a steady increase in the FAD oxidation state even below that value. This means that the FAD reduction state is a sensitive marker that already indicates metabolic restrictions prior to functional restrictions (decrease in ATP availability). Another consequence of cxII-inhibition is a shift in metabolite concentrations. The simulations predict a steady increase in succinate and a decrease in citrate and α-ketoglutarate concentrations. This is accompanied by an increase in lactate once the ATP-content decreases. These model-based findings are in agreement with (Kajimoto et al. 2014), where metabolic shifts associated with propofol administration were measured.

### Effects of propofol on hippocampal gamma oscillations and metabolism

**Fig. 5:**
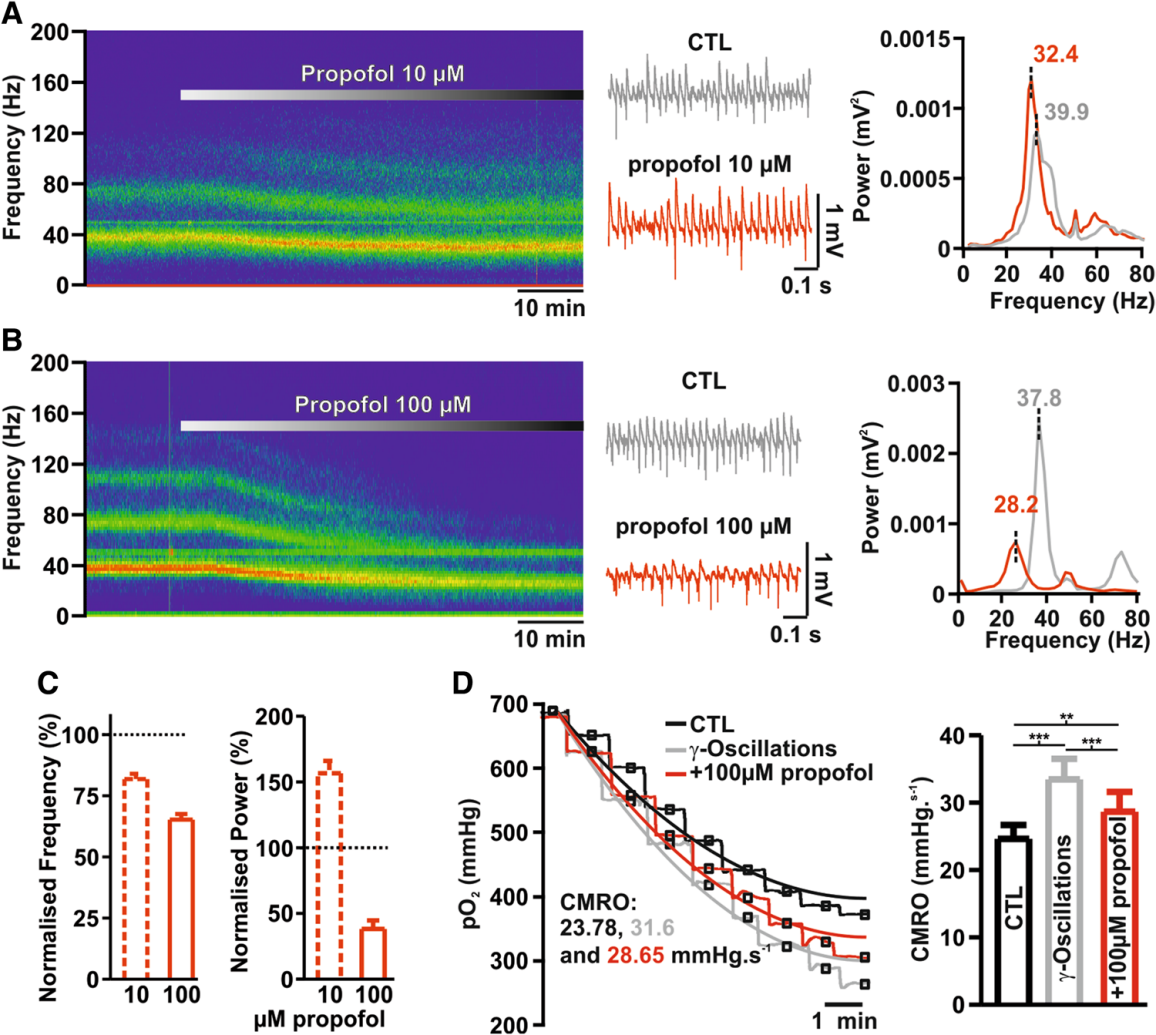
Effects of propofol on gamma oscillations and changes in CMRO_2_. **a, b** Exemplary sonograms, field potential traces and power spectra of experiments concerning effects of propofol on gamma oscillations. Gamma-oscillations were induced by simultaneous application of acetylcholine and physostigmine. The application of 10 µM propofol (**a**) was associated with increase in gamma power and decrease in frequency while under 100 µM (**b**) both power and frequency markedly decreased (gray control and red propofol). **c** Normalized changes in power and frequency of gamma oscillations under 10 or 100 µM propofol. **d** Left: Examples traces of 20 µm O_2_-steps measured under control conditions (CTL, i.e. before induction of gamma-oscillations, black traces), during gamma oscillations (gray traces) and during gamma-oscillations and treatment with 100 µM Propofol (red traces). Using the reaction–diffusion model, pO_2_-levels at each depth in the pO_2_ profile were fitted (black: CTL, gray: γ, red: γ + 100 µM Propofol) to calculate CMRO_2_. Right: statistical comparison of absolute changes in CMRO_2_ in the naïve slice, after induction of gamma-oscillations and after further treatment with propofol. All plots display Mean + SEM, significance was tested using paired *t* tests, *n.s*. non significant, *< 0.05, **0.01 and ***0.001

Finally, we tested whether the presence of propofol in concentrations leading to changes in energy metabolism might limit network activity. To this end, we studied the effects of propofol during gamma oscillations, a high activity state characterized by synchronous cellular firing and high energy demand (Huchzermeyer et al. 2013, see Fig. 5). We induced the network oscillations by pharmacological application of acetylcholine (10 µM) and physostigmine (2 µM) for ~ 90 min until stable oscillatory activity was observed (no further changes in frequency or power in online sonogram and power spectra), (see also Schneider et al. 2017). Low concentrations of propofol (10 µM) significantly increased gamma power (0.0015 vs. 0.0023 mV^2^ for control and after treatment, respectively, *p* = 0.005, *n* = 15, paired *t* test) and decrease peak frequency (from 38.4 in the control to 31.4 Hz after treatment, *p* < 0.001, *n* = 15, *N* = 5, Student *t* test). Contrary to this observation, the application of 100 µM Propofol strongly impaired oscillations as both power and frequency significantly decreased (power from 0.0021 to 0.0009 mV^2^ and frequency from 38.6 to 25.4 Hz for control and after 100 µM propofol, respectively, *p* < 0.001 for both, *n* = 13, *N* = 5, paired *t* test). We checked for changes in CMRO_2_ during gamma oscillations and subsequent changes after administration of 100 µM propofol by simultaneous O_2_-measurements. As expected, induction of gamma oscillations with acetylcholine and physostigmine strongly increased CMRO_2_ in hippocampal slices. Treatment with 100 µM propofol significantly decreased CMRO_2_ although not to control consumption rates as the slices still displayed oscillations (from 24.61 ± 2.0 in control to 33.44 ± 3.0 during gamma and to 28.65 ± 2.9 mmHg/s after propofol, *p* = 0.002 and 0.002 respectively, *n* = 12, *N* = 5, Wilcoxon).

## Discussion

### High concentrations of propofol affect synaptic transmission and decrease CMRO_2_

Our study shows that propofol at higher concentrations has a direct impact on the energy metabolism of neuronal cells. The effect of propofol on neuronal energy metabolism is (at least) twofold: First, propofol acts on synaptic and electrophysiological processes leading to a reduction in energy demand. Second, propofol reduces ATP availability by inhibition of cxII of the RC.

The changes observed in PS and PPR in our experiments confirm previous findings of an inhibition of synaptic transmission in the hippocampal formation (decrease in orthodromic PS) and decrease in transmitter release probability (decrease in PPR) associated with propofol (Higuchi et al. 2003; Wakita et al. 2013; Bademosi et al. 2018). The antidromic PS in area CA3 did not significantly change by propofol excluding relevant effects in presynaptic axonal transmission and voltage-dependent Na^+^-channels as stated previously (see Otoom and Hasan 2004). The reduced synaptic transmission directly impacts on neuronal energy metabolism as under normal conditions a significant amount of neuronal ATP consumption is accounted for by synaptic processes (Liotta et al. 2012). Decreased ATP demand leads to a decrease in F_0_F_1_-ATPase activity and reduces the backflow of protons across the mitochondrial membrane used for ATP production. This causes an increase in the mitochondrial membrane potential which inhibits complex IV of the RC and reduces CMRO_2_ (Berndt et al. 2015). To check whether the reported action of propofol as GABA_A_-agonist (Shin et al. 2018) can explain the observed changes in CMRO_2_, we tested if propofol has similar effects on CMRO_2_ after complete blockade of the GABAergic transmission., with bicuculline and under complete blockade of glutamatergic and GABaergic transmission using a cocktail containing bicuculline, AP-5 and CNQX. Under both conditions, propofol decreased CMRO_2_ showing that propofol effects on neuronal respiration are not limited to effects on the synaptic transmission. Interestingly, the cocktail completely blocked the orthodromic PS in CA3 (data not shown), thus generating a stronger decrease in synaptic transmission than 100 µM propofol, but contrary to propofol it did not significantly reduce CMRO_2_ under basal conditions.

### FAD-signal: computer simulations predict cxII inhibition and empirical confirmation

Changes in CMRO_2_ reflect the oxidative energy metabolism of the brain but this measure alone does not allow to discriminate whether CMRO_2_ is decreased due to limitations in the respiratory capacity or due to a decreased energy demand. FAD-autofluorescence allows a direct measurement of changes in mitochondrial redox state (Scholz et al. 1969; Rösner et al. 2016) and can be used to assess the metabolic state of neuronal tissue (Berndt et al. 2015). Compared to NAD(P)H-imaging, the FAD-signal is originating only in mitochondria and its recording is less phototoxic because lower energy is applied to the tissue (Scholz et al. 1969; Rösner et al. 2013). Since 100 µM propofol reduced local field potentials in our preparation (Fig. 1), our data support in part the simple interpretation of a mere reduction in CMRO_2_ due to the lowered energy demand. However, simulations of a decrease in CMRO_2_ due to a decrease in ATP-consumption alone (Fig. 1a) predict a FAD-reduction (decrease in signal since only oxidized FAD generates autofluorescence). Contrarily, the measured FAD during propofol administration showed an increase in FAD autofluorescence (Fig. 2). As it has been suggested that propofol can directly impact the RC, we conducted simulations to identify possible molecular target of propofol. We simulated the effect of PDHC, cxI, cxII and cxIII-inhibition on mitochondrial FAD redox state and showed (Fig. 4) that only cxII-inhibition can account for the observed changes. This is in line with the findings of Kajimoto et al. 2014, who showed in vivo that propofol impedes the electron flow through the RC in a coenzyme Q-dependent manner. Importantly, inhibition of cxI and cxIII leads to a strong reduction in FAD (see, Fig. 4a), while cxII inhibition leads to an oxidation shift (Figs. 2, 3a, 4a). This is because cxII is also part of the CAC (succinate dehydrogenase) and its blockade leads to oxidation in mitochondrial redox state (Berndt et al. 2012).

To confirm the predicted decrease of cxII activity by propofol administration, we simulated the change in mitochondrial redox state associated with a sudden increase in energy demand; as such stimulus-induced responses can be used to test the metabolic state of the system (Rösner et al. 2016). The simulations predicted a higher peak and diminished undershoot with cxII-inhibition compared to the uninhibited case (Fig. 2). This shift in the FAD-transients was indeed observed when 2 s tetani (20 Hz) were applied to the slice under propofol. Comparing model predictions with experimental data confirmed the changes in RC to be associated with a direct cxII-inhibition. Besides the RC, the simulation of PDHC-inhibition also was associated with FAD-oxidation as its inhibition limits the substrate availability for the CAC similar to cxII inhibition. Indeed the PDHC was also identified as target of propofol by Kajimoto et al. (2014).

Another important aspect is the degree of cxII-inhibition achieved at propofol concentrations used in this study. As shown in Fig. 4, up to 85–90% cxII-inhibition, there is no decrease in CMRO_2_ at the resting state, while FAD reduction state shifts right from the start with a steep increase at about 70%. If cxII-inhibition was above 80%, the decrease in CMRO_2_ observed would be a metabolic effect rather than a consequence of reduced energy demand, rendering the reduced activity to be a consequence of energy depletion.

### Implications for neuronal homeostasis, effect on gamma oscillations and translational aspects

It remains unclear if the changes observed in mitochondrial function after application of 100 µM propofol may elicit functional changes in the neuronal tissue. Figure 2e shows that the [K^+^]_o_ recovery to baseline after tetanic stimulation is reduced during propofol administration suggesting metabolic impairment despite reduced excitability. To test whether these limitations are important under basal conditions, we performed experiments with pharmacologically induced gamma oscillations (Fig. 5). If propofol-induced cxII inhibition limits RC activity under basal conditions, CMRO_2_ could not be increased beyond this value. However, propofol in the presence of gamma oscillations was accompanied by an increase of ~ 15% in CMRO_2_ (Fig. 5) omitting the possibility that propofol-induced inhibition of the RC limits CMRO_2_ under basal conditions.

Interestingly, gamma oscillations are increasing in power and reducing in frequency by 10 µM propofol which did not generate significant changes in PS-Amplitude, PPR, CMRO_2_ or FAD-autofluorescence. Lowering frequency and power enhancement of EGG low frequent bands is typically observed under light general anesthesia (Brown et al. 2010) and power increase of gamma oscillations was also observed during propofol-sedation in humans (Saxena et al. 2013).

Furthermore, the application of 100 µM propofol impaired gamma frequency and power with significant changes in CMRO_2_ and FAD, a metabolic and electrophysiological situation similar to deep anesthesia (Brown et al. 2010). As deep anesthesia (i.e. general anesthesia characterized by burst suppression or isoelectric prefrontal EEG) is related to poor postoperative outcome and neurological complications (Fritz et al. 2016; Radtke et al. 2013; Soehle et al. 2015a, b; Steinmetz et al. 2010) and since gamma oscillations are cognitive relevant network oscillations, our data suggest that propofol in high concentrations may impair processes related to learning and memory by influencing both the synaptic transmission and the mitochondrial function of neurons.

**Fig. 6:**
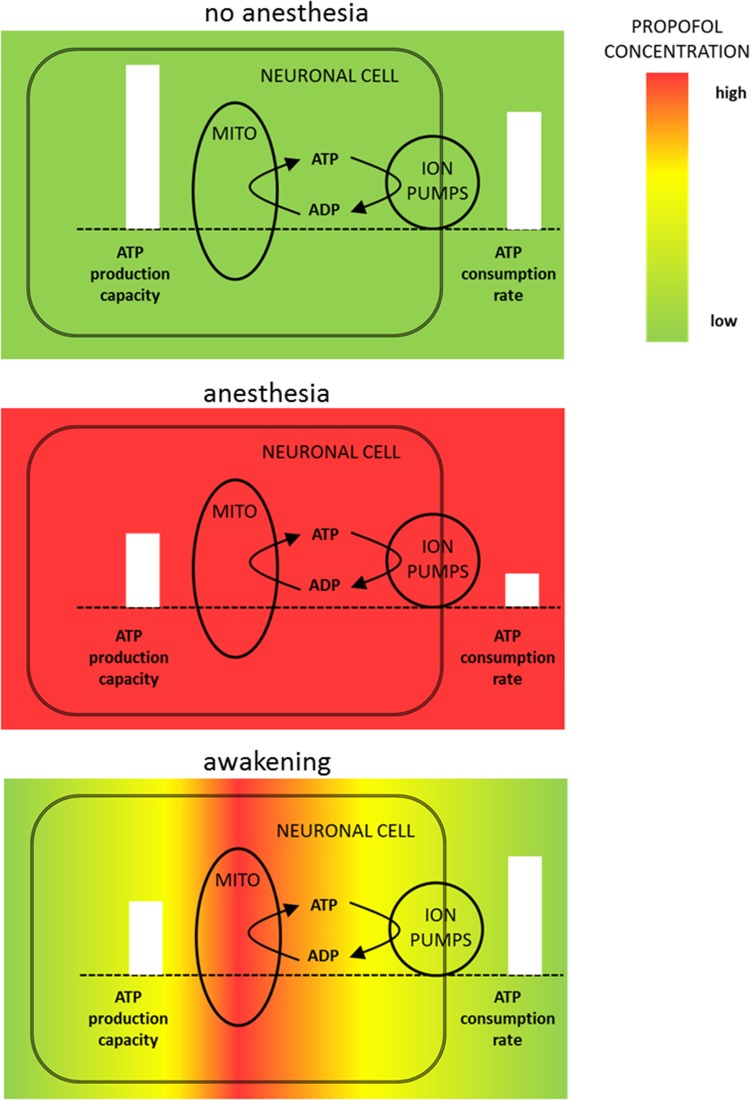
Illustration of the effects of propofol on neuronal functionality during and after anesthesia. **a** Under normal conditions neuronal ATP production capacity exceeds ATP demand ensuring demand matching energy supply. **b** During anesthesia plasma propofol levels are high and propofol reduces neuronal energy consumption by inhibition of electrophysiological processes. Simultaneously, propofol embeds in the mitochondrial membrane and reduces ATP production capacity by inhibiting cxII of the RC. The net effect is a reduced ATP demand and a reduced ATP production capacity, but demand matching energy supply is not impaired. **c** During the washout phase plasma propofol concentrations decline and the propofol effect on electrophysiology is abolished. However, due to its lipophilicity propofol is retained in the mitochondrial membrane and the ATP production capacity remains reduced leading to an energy supply demand mismatch

Our studies make plausible why propofol may exert its toxic effects on neurons, especially during the awaking period. Propofol is a very lipophilic drug which displays multi-compartment pharmacokinetics, i.e. significant elimination occurs before distribution equilibrium. During propofol administration, plasma propofol is high and equilibrates with propofol levels in the tissue. During anesthesia, propofol inhibits electrophysiological processes, thus limiting ATP demand and at the same time impairing cxII of the RC decreasing ATP supply capacity, but only to an extent where the reduced ATP demand can still be satisfied. Depending on the rate of elimination from the blood (activity of the liver, abrupt or slow cessation of propofol infusion (Kazama et al. 1998)) the local concentrations of propofol in the brain tissue will be different with lowest concentration in the blood and highest concentrations inside the cells (Soehle et al. 2015a, b). This concentration gradient of propofol may imply that cxII, located in the mitochondrial membrane, still experiences high propofol concentration, whilst synaptic and electrophysiological processes in the periphery of the neuron already experience low propofol concentrations (see Fig. 6). Consequently, the propofol-dependent inhibition of ATP supply will continue, although the ATP demand has regained full strength, resulting in a transient energetic imbalance.

Concerning the propofol inhibition of cxII in the brain of patients it is hard to compare the concentration used in the in vitro setup with the concentrations used in the clinical setting. In vivo, propofol is transported through the blood to the neurons and is degraded by the liver, while it has to diffuse through the slice in the in vitro setting but is not degraded. Furthermore, the uptake kinetics into the neuronal cells is unknown and, therefore, the extracellular concentration (if it was known) cannot be related to an intracellular concentration. Finally, even if the intracellular concentration was known, the inhibition constant for cxII is not. For these reasons, it is difficult to relate the in vivo and in vitro concentrations to a cxII-inhibition.

## Summary

We investigate the metabolic effects of propofol administration on neuronal energy metabolism by combining measurements of partial oxygen pressures in neuronal slices, electrophysiological recordings and simultaneous imaging of flavin-adenine-dinucleotide (FAD) fluorescence with a validated mathematic model of neuronal energy metabolism. Our study demonstrates that the impairment in neuronal function by propofol is not only due to the effects on the electrophysiology of neuronal tissue, but may also be the result of a direct inhibition of cxII of the RC.

## Electronic supplementary material

Below is the link to the electronic supplementary material.

Suppl. Fig. 1. Control experiments concerning possible effects of DSMO on FAD, electrophysiology and oxygen consumption. A. Sample recording of simultaneous FAD (trace in the top), f.p. (not shown), [K^+^]_o_ (trace in the middle) and pO_2_ (trace in the bottom). DMSO (0.1%) was applied to check possible effects in metabolism and electrophysiology. B. Plot of peak and undershoot of recorded stimulus-induced FAD-transients (gray: CTL and black: DMSO). C. Exemplary population spike in CA3 during control (gray) and DMSO (black). Analysis of fEPSPs, PPR, stimulus-induced K^+^-rises and pO_2_-decays did not display major changes in the presence of DMSO (n=5, n.s.= non significant, paired t-test). (TIF 8565 KB)
